# Meet the Editorial Board Member

**DOI:** 10.2174/1570159X2213240606161530

**Published:** 2024-06-06

**Authors:** R. Bakalova

**Affiliations:** 1 National Institute of Radiological Sciences (NIRS), Chiba, Japan

   Dr. Bakalova obtained a doctorate (PhD) degree in Biochemistry & Biophysics from the Bulgarian Academy of Sciences, as well as a doctorate in science (DSci) degree in Biological Sciences from the Sofia University “St. Kliment Ohridski”, Bulgaria. She has over 180 scientific publications and 12 Japanese patents. The main scientific interests of Dr. Bakalova are in the field of molecular imaging, molecular probe design, drug delivery systems, and nanomedicine. Recent studies focus on redoximaging and image-guided redox-modulation in cancer, as well as on nano-theranostics for cancer therapy.
